# Editorial: Discourse, Conversation and Argumentation: Theoretical Perspectives and Innovative Empirical Studies—Volume I

**DOI:** 10.3389/fpsyg.2021.775053

**Published:** 2021-10-22

**Authors:** Francesco Arcidiacono, Antonio Bova, Carlo Galimberti, Lise Haddouk

**Affiliations:** ^1^Research Department, Haute Ecole Pédagogique – Cantons de Berne Francophone, Jura et Neuchâtel, Biel/Bienne, Switzerland; ^2^Department of Psychology, Catholic University of the Sacred Heart, Milan, Italy; ^3^Department of Psychology, Université de Rouen, Mont-Saint-Aignan, France

**Keywords:** argumentation, communicative interactions, conversation, discourse, psychology

Thinking about how we participate in an interaction through verbal and non-verbal exchanges is a way to focus on explicit and implicit norms, personal and collective preferences, subjective and interpersonal theories, and social processes of construction of meaning that characterize our everyday communicative interactions. Although discursive, conversational, and argumentative interactions play an essential role in our lives, there is no integrated area of psychological research on these types of communicative interactions. A wide variety of works is available concerning the focus on the different roles played by social actors within the interactions (symmetric-asymmetric, protagonist-antagonist, and teacher-learner), as well as the interest for the constitutive (emotional, motivational, and cognitive) aspects of the interactions or developmental factors (skills, competences, and knowledge). However, research on these topics is conducted in a number of separate research communities that are spread across disciplines and have only limited intertwinements. This Research Topic intends to offer a dialogical platform for sharing studies on discourse, conversation, and argumentation from a psychological perspective. Different strands were bridged together to reach two main goals:

1. to explore novel and promising theoretical perspectives to study discursive, conversational, and argumentative interactions from a psychological perspective;
2. and provide an extensive platform of the latest innovative research investigating interactions between individuals, groups, and institutions.

The Research Topic provided an opportunity for researchers working in different international and psychological contexts to draw together their work within a common forum. Through their contributions, authors provided state-of-the-art of collective evidences in psychological research on discourse, conversation, and argumentation. They were able to place emphasis on opportunities for mutual awareness and integration by proposing a series of high-quality original research papers, reviews, and conceptual analyses on topics dealing with the above-mentioned issues. The contributions are focused on psychological perspectives on interactions and empirically supported approaches to analyze them. This panel of respectful researchers contributed to render the Research Topic particularly timely and open to colleagues who are continually exposed to nearly limitless sectorial approaches. In this vein, we hope that the contributions can be challenging for a large scientific audience to support integrated psychological research on discursive, conversational, and argumentative interactions.

The paper of Koskinen et al. presents an original research offering a better understanding of the affective corollaries of social interactions during a psychophysiological experience of solving moral dilemmas. The authors compared participants with and without depression and showed their positions in expressing agreements and disagreement while solving moral dilemmas together.

Heller proposes a study on embodied displays of “doing thinking” by analyzing the epistemic and interactive functions of thinking displays in children's argumentative activities. From an interactional perspective, her paper investigates moments in which one participant in an interaction embodies displays of doing thinking as a recurring social practice serving interactive functions. The activities of joint decision-making are considered the functional argumentative setting to reach two interrelated aims: the first is to describe how multiple modalities (gaze and bodily postures) are temporally coordinated to create multimodal gestalts of doing thinking; the second one is to generate knowledge about the functions of thinking displays in children's argumentative activities.

Jiménez-Aleixandre and Brocos propose another original paper to highlight the relationship between argumentative scientific evidences and emotions. In particular, the authors focus on the exam of the ways through which emotional tensions frame the construction of arguments about vegetarian vs. omnivorous diets within a group of preservice teachers. Their investigation shows how the interactions between the group emotional tension and the evaluation of evidence drive a change toward a decision that would be emotionally acceptable for all participants.

The paper proposed by Galimberti et al. considers the direct address as a research object within the field of social psychology of communication. Their research contributes to increase our understanding of this technique by going beyond the analysis of its dramaturgical function (through the consideration of a TV series). In fact, the authors propose an integrated approach based on argumentative and conversational tools, showing that the direct address performs its dramaturgical function by impacting both diegetic and extradiegetic levels.

Another original research is proposed by Fatigante et al., who analyze how the companion participation during first oncological visits is a local and sequential accomplishment, changing from time to time in the consultation. The paper focuses on how patient's companions orient and contribute to the accomplishment of the different aims and activities at different stages of the visit as an institutional speech event. The authors refer to a multimodal analysis of turns and actions to closely examine the sequential and temporal arrangements of companions' and their co-participants' turns.

A conceptual analysis is provided by Leijen et al. to consider different understandings of inclusive education that frame current public and professional debates as well as policies and practices. The authors analyze two discourses regarding inclusive education by reconstructing the inferential configurations of the arguments of each narrative. The paper contributes to identify how the two definitions position children with and without special needs and their teachers.

Iordanou and Rapanta propose a review about a method for developing argument skills. The authors examine a particular program, the “Argue with Me” dialogue-based pedagogical approach, not only as a tool for supporting the development of argument skills but also the way of how empirical research employing the method in varying contexts provides insights into the nature of argument skills and their development, as well as the relations between argument skills and other skills or forms of understanding.

Another review (Larrain et al.) considers the relevance of deliberative education for contemporaneous democracies and citizenship, seeking to converge in a field of interlocution, calling it deliberative teaching. The paper proposes a way to increase the dialog and collaboration between the diffuse literature on argumentation and education. The authors highlight both the main theoretical and empirical gaps and challenges, as well as the possibilities to advance our knowledge and the educational impact that this integrating field could offer.

## Author Contributions

All authors listed have made a substantial, direct and intellectual contribution to the editorial, and approved it for publication.

## Conflict of Interest

The authors declare that the research was conducted in the absence of any commercial or financial relationships that could be construed as a potential conflict of interest.

## Publisher's Note

All claims expressed in this article are solely those of the authors and do not necessarily represent those of their affiliated organizations, or those of the publisher, the editors and the reviewers. Any product that may be evaluated in this article, or claim that may be made by its manufacturer, is not guaranteed or endorsed by the publisher.

